# Background Values of Soil Heavy Metals in the Huang-Huai-Hai Plain in Henan Province, China

**DOI:** 10.3390/toxics13020093

**Published:** 2025-01-26

**Authors:** Yuling Jiang, Jianhua Ma, Yuanbo Wang, Yahan Yang

**Affiliations:** 1 School of Geographic Sciences, Xinyang Normal University, Xinyang 464000, China; jiangyuling@xynu.edu.cn (Y.J.); 31043@xynu.edu.cn (Y.W.); 061128@xynu.edu.cn (Y.Y.); 2 College of Geography and Environmental Science, Henan University, Kaifeng 475004, China; 3 Key Laboratory of Geospatial Technology for the Middle and Lower Yellow River Regions (Henan University), Ministry of Education, Kaifeng 475004, China

**Keywords:** Huang-Huai-Hai plain, Henan province, soil heavy metals, background values, regional difference

## Abstract

Due to the continuous lack of specific background values (BVs) for soil heavy metals in the Huang-Huai-Hai Plain in Henan province (HPHP), many researchers have used soil heavy BVs specific to Henan Province (HP) or Fluvisols of China (FC) as reference criteria to assess soil heavy metal pollution. However, spatial differences in the soil heavy metal BVs between HPHP, HP, and FC, as well as within the HPHP, remain uncertain, affecting the reliability of evaluation results. A total of 897 surface soil samples were collected from the HPHP, with 336 and 561 samples collected from the southern and northern parts of the Shaying River, respectively. According to the obtained results, the BVs of soil Hg, As, Cd, Cr, Pb, Cu, Zn, and Ni in the HPHP were 0.064, 6.67, 0.129, 53.24, 19.67, 22.87, 64.00, and 26.25 mg·kg^−1^, respectively. The BVs of soil Hg and Cd were higher than those in HP, Fluvisols in Henan Province, and FC, showing strong and extremely strong levels. The BVs of other soil heavy metals exhibited slight differences from the reference BVs. On the other hand, the BVs of soil Hg, As, Cd, Cr, Pb, Cu, Zn, and Ni were 0.066, 4.11, 0.130, 56.72, 20.97, 23.31, 59.21, and 24.03 mg·kg^−1^ in the southern part and 0.061, 7.45, 0.129, 51.92, 18.96, 22.72, 66.96, and 27.16 mg·kg^−1^ in the northern part of the Shaying River, respectively. In addition, there were no significant differences in the Hg and Cd BVs between the two parts. Cu BVs in the southern part were significantly higher than those observed in the northern part, while the As, Zn, and Ni BVs in the northern part were significantly higher than those revealed in the southern part. In contrast, the Cr and Pb BVs in the northern part were significantly lower than those observed in the southern part.

## 1. Introduction

The background values (BVs) of soil heavy metals are important soil and earth chemical data, providing further insights into the evaluation of soil heavy metal pollution. In other words, the BVs of soil element are the characteristics of soil parent quality in areas that are not affected by human activities [1,2,3]. However, in recent years, soils have been considerably affected by human activities due to increasing urban, agricultural, and industrial development. Indeed, even deep soil layers have become affected to some extent by pollutants through the leaching process, making it difficult to define strict element BVs [4]. In this context, the baseline value concept of soil elements was proposed in the 1990s, referring to the content range of soil elements in a certain area and at a specific time under natural and anthropogenic conditions (excluding point-like pollution sources) [4,5,6]. In 1996, the International Organization for Standardization (ISO) referred to the above soil BVs as natural BVs and reference values as BVs, which has been accepted by many scholars [2,4,7]. The concept of soil heavy metal BV described in this study was based on the BV superimposed on the natural BV influenced by human activities rather than the natural BV, which is conceptually the same as the reference value.

The BVs of soil elements were studied in China as early as 1986~1990, providing soil heavy metal BVs in a layer of the main soil types, provincial administrative regions, and main parent material types in China [8]. However, some deviations may occur in the evaluation results of soil heavy metal pollution using specific BVs in provincial administrative regions at different geographical units due to the vast areas, complex geological conditions, and diverse soil types of these regions. For example, the western and southern parts of Henan province are mountainous, accounting for 44% of the province’s surface area. In addition, soil types in these areas consist mainly of Fluvisols, Cambisols, Luvisols, Planosols, Anthrosols, Regosols, and Vertisols. The Huang-Huai-Hai plain is located in the eastern part of Henan province, covering 45% of the province’s area and consisting of several soil types, including Fluvisols, Cambisols, Planosols, Anthrosols, and Regosols. Therefore, it is not appropriate to use the soil heavy metal BVs in Henan Province as reference standards to evaluate the soil heavy metal pollution in the Huang-Huai-Hai Plain of Henan Province (HPHP).

As a part of the national key scientific and technological project (75 Plan), the Henan Province Soil Survey Bureau conducted a survey on soils in Henan Province, providing BVs of heavy metals in Fluvisols specific to Henan Province [9]. However, the Cu and Zn BVs in the Fluvisols of Henan Province were not considered in the survey. Besides Fluvisols in the HPHP, there are several other soil types, of which Planosols are the most abundant. Large areas are also covered by the Fluvisol type in the Luoyang and Sanmenxia basins in the western part of Henan Province, as well as the southwestern part of the Nanyang basin, suggesting spatial differences in the soil heavy metal BVs of the Fluvisol types between Henan Province and the Huang-Huai-Hai Plain. In addition, the BVs in the Fluvisols of Henan Province investigated at that time were only published in the Henan Soil book [9], which has little academic influence, making its application greatly limited.

In this context, many scholars have used the soil element BVs of Henan Province [8] as reference standards to carry out soil heavy metal pollution assessments in the Henan Plain [10,11,12,13,14]. In addition, some scholars have also used the Fluvisols BVs of China [8] as reference standards to assess soil heavy metal pollution [15,16,17,18,19,20,21], since the Fluvisol type is widespread in different natural areas in China. However, the BVs of Fluvisols in China cannot comprehensively represent the soil BVs in the HPHP. Although some scholars have used the average soil heavy metal contents in control soil samples near the study areas to evaluate soil pollution in some parts of the Henan Plain [22,23,24,25], the control soil sample size and sampling range were limited, which cannot accurately represent the soil BVs of the study area, thereby affecting the reliability of the evaluation results.

Indeed, investigating the spatial differences between the BVs of soil heavy metals in the HPHP and those of the Fluvisols of China and the Henan Province is of great significance for assessing soil heavy metal pollution. Heavy metal contamination levels influence the mobility of these metals within soil systems [26]. Indeed, the HPHP is large, spanning the warm temperate zone and the northern subtropical zone of China, exhibiting obvious spatial differences in the soil types. Therefore, whether there are differences in the BVs of soil heavy metals within the plain is also a scientific question to be answered.

In this context, soil surface samples were collected in this study from the HPHP and analyzed for Hg, Cd, Pb, As, Cu, Zn, Cr, and Ni contents to determine their relative BVs according to relevant technical requirements. The main objective of the present study was to provide more accurate background data for an effective assessment of heavy metal pollution in the soil in Henan province, which is of great significance for protecting the soil environment and maintaining food security.

## 2. Material and Methods

### 2.1. Physical Geography of the Study Area

**Figure 1:**
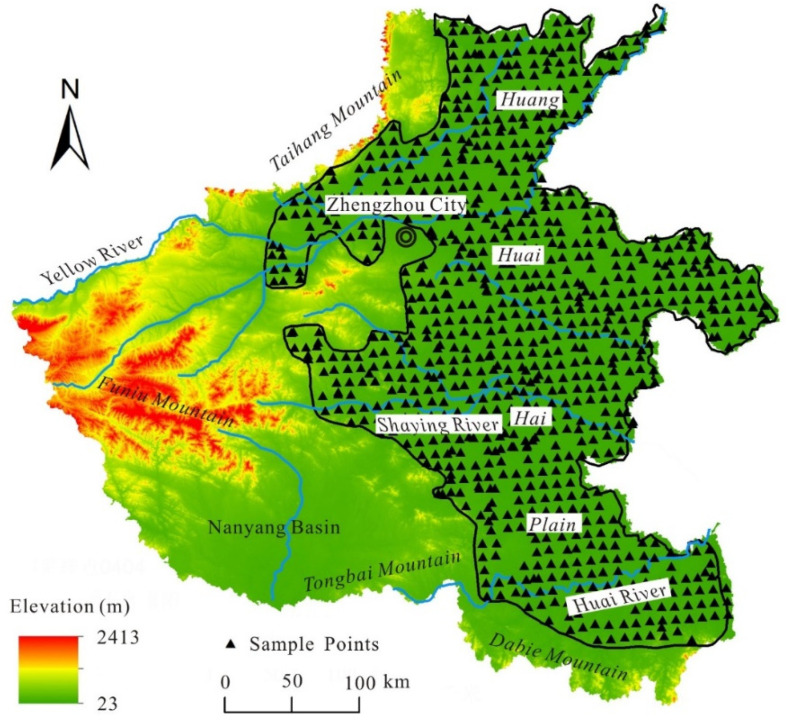
Geographic location of the study area and soil sampling points.

The HPHP is located in the western part of the third terrain ladder in China. The western and southern parts are bounded by Taihang, Funiu, Tongbai, and Dabie Mountain foothills at 200 m above sea level, while the eastern part is bounded by Henan Province (Figure 1). The surface area of the HPHP is about 85 × 104 km^2^, accounting for 53% of the land area of Henan Province. From a geomorphological point of view, the area is part of the North China Plain, with an altitude range of 40–100 m. In addition, the northern alluvial part of the study area is bounded by the mainstream of the Yellow River and Haihe River, most of which belong to the Haihe River Basin. Whereas the southern alluvial plains and lake plains are bounded by the Yellow River and the Huai River, belonging to the Huai River Basin. The climate in the study area is characterized by a north–south transition. The northern mainstream of the Huai River belongs to the warm temperate monsoon climate, with average annual temperature and average annual precipitation ranges of 13.5–14.6 °C and 530–750 mm, respectively. The southern part of the study area belongs to the northern subtropical monsoon climate, with average annual temperature and average annual precipitation ranges of 14.5–15.2 °C and 850–900 mm, respectively.

Besides the construction land, almost the entire HPHP is agricultural land, where wheat/corn and wheat/rice are the main crops, covering large areas of the northern and southern parts of the mainstream of the Huai River, respectively. On the other hand, Fluvisols, Planosols, Cambisols, Anthrosols, and Vertisols are the main soil types in the study area [27], of which the Fluvisol type is the most abundant soil type, covering about 65% of the total area. In addition, the Fluvisol type is distributed mainly in the northern part of the mainstream of the Shaying River. This soil type is characterized by deep soil profiles, light texture, and a pH range of 7.5–8.5, with the presence of flooded alluvial Cambisols on the western edge of the study area. Planosols are widespread in the southern area of the mainstream of the Shaying River, covering about 20% of the total area. It is characterized by a finer texture and pH of about 7.0. In addition, some areas exhibit complex soil types, including the Regosol and Luvisol types. Planosols are mainly distributed in the southern part of the mainstream of the Huai River, where the Fluvisol and Regosol types are present, covering a small area and exhibiting pH values of about 6.5.

### 2.2. Soil Sampling

The 10 km × 10 km grid method was used in this study to collect soil samples from uniform sampling points. The soil samples were collected near the center of the grid point. In order to avoid the interference of human activities with the soil analysis results, sampling points were more than 5 km away from the city, more than 2 km away from township settlements, traffic arteries, and industrial enterprises, more than 1 km away from villages, and more than 200 m away from farmland roads and ditches. In this study, a total of 897 soil samples were collected. The spatial distribution of sampling sites is shown in Figure 1.

In total, 5 soil samples were first collected from the 0–20 cm soil layer at each 10 m × 10 m sampling grid using the quincunx method, then mixed and separated from plant residues and stones according to the quartering method to obtain a 1 kg composite soil sample. The collected composite soil samples were air-dried in the laboratory at room temperature, crushed with wooden sticks, sieved using 100-mesh and 10-mesh nylon sieves, and well-mixed for further analysis.

### 2.3. Determination of Soil Heavy Metal Concentrations

The collected soil samples were first digested with HNO_3_ ($\rho_{{HNO}_{3}}=1.42\;g/mL$)-HF ($\rho_{HF}=1.49\;g/mL$)-HClO_4_ ($\rho_{{HClO}_{4}}=1.68\;g/mL$) [28], then the Ni, Pb, Cd, Cr, Cu, and Zn contents were determined using an inductively coupled plasma mass spectrometer (XSeries-2 ICP-MS, Thermo Fisher Scientific, 81 Wyman Street, Waltham, MA, USA) [29]. The Hg and As concentrations were determined by aqua regia digestion ($\rho_{HCl}\;=\frac{\;1.19\;g}{mL};\rho_{{HNO}_{3}}=1.42\;g/mL$) and an atomic fluorescence spectrometer (AFS-3100, Beijing Haiguang Instrument Co., Ltd., Beijing, China) [30,31]. The reagents employed were of analytical grade. Besides the deionized water used in the analysis, all the reagents used were of excellent quality grades (Tables S1 and S2). Furthermore, the national standard soil reference material (GSS-2), repeat analyses, and blank analyses were considered the analytical process to assess the accuracies of the measurement results. The standard soil sample (GSS-2) was purchased from the Center of National Standard Reference Material of China. The recovery rates of soil heavy metal ranged from 85 to 108%, while the relative errors of parallel samples varied from 5 to 25%. The detection and quantification limits were as follows: 0.002 mg/kg and 0.007 mg/kg for Hg, 0.001 mg/kg and 0.003 mg/kg for As, 0.0003 mg/kg and 0.0009 mg/kg for Cd, 0.004 mg/kg and 0.014 mg/kg for Cr, 0.004 mg/kg and 0.015 mg/kg for Pb, 0.004 mg/kg and 0.015 mg/kg for Cu, 0.07 mg/kg and 0.25 mg/kg for Zn, and 0.004 mg/kg and 0.015 mg/kg for Ni.

### 2.4. Determination of the Background Soil Heavy Metal Values

At present, there is no universal method for determining the BVs of soil elements. However, the basic determination method consists of performing a data distribution test to remove the abnormal soil heavy metal content values at a 95% confidence level [3,6,8,32,33].

#### 2.4.1. Outlier Test

There are many methods to detect outliers in the original soil heavy metal data, including the arithmetic mean method (Mean ± SD) [1,32,33,34], regression analysis [32,34], Tukey’s test [6,33,35,36,37], and Grubbs and Dixon method [38].

However, since the maximum sample size of the critical value data in GB/T 4883-2008 [38] is only 100, which is considerably lower than that considered in the present study, the Grubbs and Dixon method is not suitable for this study. On the other hand, the arithmetic mean method revealed a higher number of anomalous soil heavy metal values than that obtained using Tukey’s test. The numbers of Hg, Cd, and Pb abnormal values in the soils of HPHP were 90, 79, and 36 using Tukey’s test (cyclic test until no abnormal values appear) and 193, 251, and 119 using the arithmetic mean method, respectively. The numbers of the Hg, Cd, and Pb abnormal values, obtained using the arithmetic mean method, were 2.14, 3.18, and 3.31 times higher, respectively, than those obtained using Tukey’s test. Therefore, in order to optimize the accuracies of the background soil heavy metal values, the arithmetic mean method was used in this study to test and remove outliers from the soil heavy metal data.

#### 2.4.2. Data Distribution Test

There are several methods of assessing the distribution of data and further improving the accuracies of soil heavy metal BVs. In this study, the skewness *Z* score (*Z_s_*), kurtosis *Z* score (*Z_k_*), single sample Kolmogorov–Smirnov nonparametric test, and Q-Q plot method were used to perform the distribution tests. The *Z_s_* and *Z_k_* were calculated according to the following formulas:

(1) $$Z_{s}=\frac{\left|S-0\right|}{S_{s}},Z_{k}=\frac{\left|K-0\right|}{S_{k}}$$

where S and K denote the skewness and kurtosis of the heavy metal content values, respectively, and *S_s_* and *S_k_* denote the standard deviations of the skewness and kurtosis values, respectively. Observational data or their logarithmic *Z_s_* and *Z_k_* values of ±1.96 (α = 0.05) indicate normal or lognormal distribution. Otherwise, observation data or their logarithmic *Z_s_* and *Z_k_* values follow skewed distributions [39].

On the other hand, the data distribution is considered normal or log-normal if the quantiles of the measured soil heavy metal data or its log-transformed data are close to a straight normal line of the quantile scatter plot (Q-Q plot). Otherwise, the data distribution is considered skewed.

The one-sample K-S non-parametric test is used to infer whether the overall data distribution follows a normal distribution. The data distribution is considered normal at the α > 0.05 significance level. Otherwise, the assumption of a normal distribution is rejected.

In this study, the data distribution was considered normal when three of the above-mentioned tests (*Z_s_*, *Z_k_*, K-S, and Q-Q) demonstrated normal distributions of soil heavy metal data. Otherwise, the data distribution was considered abnormal.

#### 2.4.3. Methods for Characterizing Background Values

In this study, when the soil heavy metal data are normally distributed, the BVs are expressed as the arithmetic mean (AM), and the 95% confidence range of the data is defined as AM ± 2SD. If the soil heavy metal data are log-normally distributed, the background values are expressed as the geometric mean (GM), and the 95% confidence range of the data is defined as GM × GSD^2^ and GM/GSD^2^ [2,3,6,8,40]. On the other hand, the median (Med) was used in this study to determine the skewness of the data distribution, and Med ± 2MAD (median absolute deviation) was used to determine the 95% confidence range of the background soil heavy metal values [3,6,36,37]. The MAD value was calculated in this study as follows:

(2) $$\mathrm{MAD}=\mathrm{median}\;[\left|x_{i}-\mathrm{median}(X)\right|]$$

where x_i_ denotes the *i*th data in dataset X and median (X) denotes the median of dataset X. The lower line of the 95% confidence interval was represented by a numerical value corresponding to the accumulation frequency of 5% of the data when the Med–2MAD values were lower than the Med values [41].

The outlier and data distribution tests, as well as the BVs calculation of the soil heavy metals content data, were performed using IBM SPSS Statistics 21.

## 3. Results and Discussion

### 3.1. Descriptive Statistics and Distribution of Raw Data

The AM values of the Hg, As, Cd, Cr, Pb, Cu, Zn, and Ni contents in the collected soil samples of the HPHP were 0.108, 6.81, 0.171, 58.45, 20.93, 33.08, 86.62, and 27.29 mg·kg^−1^, respectively. These AM values did not exceed the risk screening values at pH > 7.5 of the National Farmland Soil Quality Standard of China [42], even though some sampling sites exhibited higher Cu, Zn, As, and Cr contents than the standard values by 3.68, 1.45, 0.45, and 0.11%, respectively. The As, Cr, Pb, and Ni contents in the soil samples exhibited moderate spatial variations, showing a coefficient of variation (CV) range of 20–50%. However, the spatial variations in the Cu and Zn contents were strong, while those of Hg and Cd contents were very strong, showing coefficient of variation ranges of 50–100% and >100%, respectively. This finding indicated that the spatial variations in Hg and Cd contents in HPHP were obvious, suggesting different degrees of anthropogenic heavy metal inputs. Meanwhile, the distributions of the As, Cr, Pb, and Ni contents were relatively uniform.

**Figure 2:**
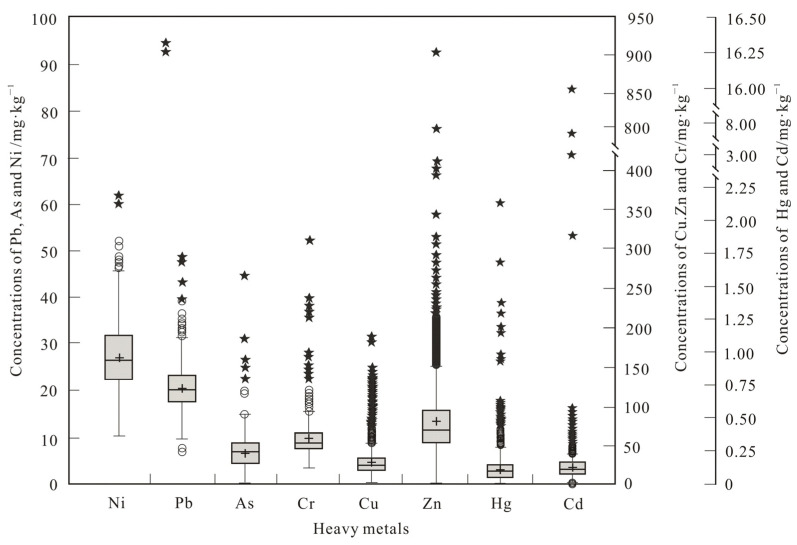
Box plot of the original soil heavy metal content data.

The box plots of the raw soil heavy metal data (Figure 2) showed a certain number of mild (○) and extreme outliers (★) in each soil heavy metal data category. Indeed, most outliers were observed at the upper limit of the scale, especially for Cu, Zn, Cd, and Hg. The median lines of the majority of heavy metal contents were plotted in the lower part of the box and do not coincide with their arithmetic means, suggesting that the heavy metal data followed abnormal distributions.

According to the Z_s_, Z_k_, K-S, and Q-Q test results, the original soil heavy metal content data followed skew distributions. In addition, the Ni data exhibited an abnormal distribution following log-transformation.

### 3.2. Statistics and Distribution of the Soil Heavy Metal Contents After Removal of Outliers

According to the results obtained using the arithmetic mean method, 193, 49, 251, 178, 119, 284, 447, and 144 outliers were observed in the Hg, As, Cd, Cr, Pb, Cu, Zn, and Ni contents’ data, accounting for 21.52, 5.46, 27.98, 19.84, 13.27, 31.66, 49.83, and 4.91% of the total number of samples, respectively. It can be seen that there were more outliers in other heavy metal content data, except for As and Ni. In addition, outliers were observed mainly in the high soil heavy metal content data, indicating that the soil Zn, Cu, Cd, and Hg contents in the Huang-Huai-Hai plain in Henan Province were considerably affected by human activities.

After removing outliers, the Hg, As, Cd, Cr, Pb, Cu, Zn, and Ni contents in HPHP were 0.070, 6.43, 0.129, 53.24, 19.77, 23.30, 64.00, and 26.25 mg·kg^−1^ (AM values), 0.054, 5.92, 0.126, 52.45, 19.56, 22.87, 62.88, and 25.74 mg·kg^−1^ (GM values). Most heavy metals exhibited higher AM values than GM values, while the median values were between the AM and GM values (Table 1). In addition, except for the soil As contents, the CV values of soil heavy metals decreased considerably after removing outliers, among which the CV values of the Hg and Cd content decreased from extremely strong variations to strong variations and moderate variations, while those of the Cu and Zn contents decreased from strong variations to slight variations. Moreover, the CV values of the Pb, Cr, and Ni contents decreased from moderate variations to slight variations.

The distribution results of the data after removing the outliers from the soil heavy metal content data are reported in Table 2. The Cd, Cr, Zn, and Ni content data followed normal distributions, while the Cu content data followed a log-normal distribution. In contrast, the Hg, As, and Pb data distributions were skewed.

### 3.3. Background Values for Soil Heavy Metals

In this study, the BVs for soil heavy metals and their 95% confidence intervals (Table 3) for the HPHP were determined using the above-mentioned methods, as well as the heavy metal contents (Table 1) and their data distribution characteristics (Table 2) after excluding outliers. The BVs for soil Hg, As, Cd, Cr, Pb, Cu, Zn, and Ni were 0.064, 6.67, 0.129, 53.24, 19.67, 22.87, 64.00, and 26.25 mg·kg^−1^, with 95% confidence intervals of 0.012–0.130, 2.75–10.59, 0.075–0.183, 35.02–71.46, 15.03–24.31, 15.66–33.39, 40.36–87.64, and 16.09–36.41 mg·kg^−1^, respectively.

(3) $$\begin{array}{l} Variability\;of\;background\;values\;\left(\%\right)\\ =\frac{background\;value\;of\;this\;study-Literature\;background\;value}{background\;value\;of\;this\;study}\times 100\% \end{array}$$

The absolute degrees of variations between the BVs of soil heavy metals were classified into four classes, namely slight (<10%), moderate (10–30%), strong (31–50%), and very strong (>51%) variations.

The obtained results showed considerable differences between the BVs of the soil Hg, Cd, and As in the HPHP and those reported in the literature (Table 4). The Hg BV was higher than that reported in the literature, showing strong to extremely strong variations. The same result was observed between the BV of soil Cd, showing variation differences. In addition, the results of the present study revealed a considerably lower As BV than those reported in the literature, showing strong to extremely strong variations. However, although positive and negative degrees of difference between the Cr, Pb, Cu, Zn, and Ni background values of the present study and those reported in the literature, weak to moderate differences were observed. These discrepancies in soil heavy metal BVs may be due to the scale of the study area, the complexity of the soil assemblage, and the sample size, requiring further in-depth investigations. Indeed, Henan Province is located between the second and third terraces of China’s landscape, where mountains, hills, plains, and a variety of parent rocks and soil-forming matrices are found. The northern boundary of the subtropical regions is extended through the south-central part of Henan Province, exhibiting considerable spatial differences in the precipitation and temperature conditions between the northern and southern parts. In addition, there are several soil types in the study area, including Cambisols, Fluvisols, Luvisols, Planosols, Anthrosols, Regosols, and Vertisols. These complex natural environmental conditions explain the considerable differences in the background values of soil heavy metals between Henan province and the Huang-Huai-Hai Plain soils. Fluvisols are intra-zonal soils, widely distributed in alluvial plains in all climatic zones (tropical, subtropical, temperate, and cold). However, the parent material and composition of Fluvisols vary considerably, explaining the differences in the background values between Fluvisols in China and those in the Henan plain. Although Fluvisols are predominant in the Henan plain, there are also areas with Planosols, Cambisols, Anthrosols, Vertisols, and Luvisols. Moreover, the Sanmenxia and Luoyang basins in western Henan and the Nanyang basin in south-western Henan also exhibit large areas with Fluvisols, explaining the differences between the BVs of heavy metals in the Fluvisols of Henan Province and those in the HPHP.

The accuracy of the BVs of soil elements is influenced by the sample size. The larger the sample size, the better the representation and the more reliable the BVs are. In the previous investigations, only 86 [8] and 81 [9] soil samples were considered in the determination of the BVs of soil heavy metals in Henan Province and Fluvisols of Henan Province, respectively. The small sample density may affect the accuracy of the BVs. For example, the BVs of soil heavy metals in Beijing [8] were determined using 40 soil sampling points. Chen [43] selected 120 soil samples of woodland and wasteland from 803 soil samples collected from the upper soil layer in Beijing to determine the BVs and highlighted Cd and As BVs of 0.119 and 7.09 mg·kg^−1^, which were 125 and 24.5% higher and lower than those revealed by the China Environmental Monitoring Center [8], respectively.

In addition, the BVs of soil elements have time-related characteristics. Indeed, the impacts of human activities on soil may inevitably increase over time, thereby increasing the contents of some soil heavy metals. The BVs of Hg and Cd in HPHP were higher than those in FH. Whereas the BVs of soil Cr, Pb, Cu, Zn, and Ni in HPHP were not substantially different from those in the other areas (Table 3). Therefore, it is believed that although human activities may impact the BVs of Hg and Cd in the HPHP to some extent, the impacts remain limited.

### 3.4. Analysis of Differences in the Heavy Metal Background Values of Soils in the Huang-Huai-Hai Plain in Henan Province

The BVs of soil elements are affected by many factors, including parent material types, weathering, groundwater, and human activities. As mentioned above, the HPHP is bounded by the mainstream of the Shaying River, and exhibits several soil types, of which Fluvisols and Cambisols dominate the northern part, while Planosols and Anthrosols characterize the southern part. In addition, these two parts of the Huang-Huai-Hai Plain are characterized by a spatial difference in the climate type, sources of soil parent material, degree of mineral weathering and leaching, and soil pH values. It is, therefore, necessary to investigate the differences in the soil heavy metal BVs between the northern and the southern parts of the HPHP.

In this study, 897 soil samples were collected from the HPHP, of which 336 and 561 soil samples were collected from the southern and northern parts of the Huang-Huai-Hai Plain, respectively. In addition, outlier values were first removed from the soil heavy metal data then the distribution tests were performed using the aforementioned methods to calculate the BVs for each heavy metal data in the southern and northern parts. The differences in the obtained BVs between the northern and southern parts were statistically analyzed using significance tests for differences in background values (Table 5). The *t*-test and rank sum were performed for normal/lognormal and skewed heavy metal data distributions, respectively. *p* > 0.05, *p* < 0.05, and *p* < 0.01 indicate nonsignificant, significant, and highly significant differences, respectively.

As can be seen from Table 5, the BVs of Hg, As, Cd, Cr, Pb, Cu, Zn, and Ni in the southern part of the HPHP were 0.066, 4.11, 0.130, 56.72, 20.97, 23.31, 59.21, and 24.03 mg·kg^−1^, with 95% confidence intervals of 0.003–0.144, 1.23–6.99, 0.092–0.168, 34.96–78.48, 15.98–27.68, 15.71–30.91, 41.12–85.26, and 13.63–34.07 mg·kg^−^^1^, respectively. In comparison, in the northern part, the BVs of Hg, As, Cd, Cr, Pb, Cu, Zn, and Ni were 0.061, 7.45, 0.129, 51.92, 18.96, 22.72, 66.96, and 27.16 mg·kg^−^^1^, with 95% confidence intervals of 0.003–0.119, 5.15–9.75, 0.069–0.189, 35.22–68.62, 14.04–25.60, 13.82–31.63, 45.68–97.37, and 17.04–37.28 mg·kg^−^^1^, respectively. There were no significant differences in the BVs of Hg and Cd between the southern and northern parts in HPHP. However, the BVs of Cu in the southern part were significantly higher than those observed in the northern part at *p* < 0.05, while the BVs of As, Zn, and Ni in the northern region were significantly higher than those revealed in the southern region at *p* < 0.01. In addition, the BVs of Cr and Pb in the northern part were significantly lower than those observed in the southern part at *p* < 0.01.

The reasons for the significant and highly significant differences in the BVs of soil heavy metals between the southern and northern parts of the plain in Henan province may be related to the differences in parent materials, climatic conditions, soil-forming time, properties of heavy metals, and agricultural activities between the two parts of the plain. The parent material in the north is mainly alluvium of the Yellow River, while the parent material in the south is mainly lacustrine sediments. The Hg BVs of the two parent materials were the same at 0.018 mg/kg. The BVs of Cd in the southern and northern parts were 0.059 mg·kg^−^^1^ and 0.054 mg·kg^−^^1^, respectively, showing a slight difference [9]. Gaseous mercury emitted by human activities can exhibit a large-scale migration and, subsequently, enter the soils through dry and wet depositions [44], further explaining the significant difference in the soil Hg background values between the northern and southern parts of the HPHP. According to Chen [44] and Wang [11], agricultural activities are the main anthropogenic source of Cd in soils. Tillage systems, crop types, and farmland management measures in the northern and southern areas of the HPHP were basically the same, suggesting similar agricultural activities-derived Cd amounts entering soils, thereby further supporting the lack of a significant difference in the soil Cd BVs between the northern and southern areas of the HPHP. The BVs of Pb and Cr in the Yellow River alluvial soils in Henan Province were 18.1 mg·kg^−^^1^ and 56.7 mg·kg^−^^1^, respectively, which were lower than those of the lacustrine sediments at 21.8 mg·kg^−^^1^ and 74.1 mg·kg^−^^1^, respectively [9]. The BVs of soil Pb and Cr in the northern part were significantly lower than those in the southern part under similar agricultural activities in the HPHP. Soil As has different chemical forms and valence states, and its activity is closely related to pH and oxidation-reduction (Eh) [45]. Increases in pH values, colloidal negative charges, and sulfate and sulfite contents in soil solution are conducive to As migration. Although the soil pH value of the southern area of HPHP was lower than that of the northern area, the hydrothermal conditions in the southern area are suitable. Moreover, soils in the southern area have a certain degree of iron (Fe)-rich aluminization. Anthrosol is characterized by a heavy texture, making it prone to water accumulation in rainy seasons and, consequently, promoting As leaching. Therefore, the As BV of soil surface layers in the southern region was significantly lower than that in the northern region. On the other hand, further studies are required to explain the differences in the BVs of Zn, Ni, and Cu in HPHP.

## 4. Conclusions

The BVs of Hg, As, Cd, Cr, Pb, Cu, Zn, and Ni in the Huang-Huai-Hai Plain in Henan Province were 0.064, 6.67, 0.129, 53.24, 19.67, 22.87, 64.00, and 26.25 mg·kg^−1^, respectively. The BVs of Hg in the HPHP were generally higher than those of soils in Henan Province and Fluvisols in Henan Province and China, showing strong and extremely strong differences of about 31–50% and >51%, respectively. The BV of Cd was also relatively high, showing strong variations. In comparison, the BV of As was generally lower than that of the reference area or the reference soil, exhibiting strong to extremely strong variations. Although there were positive and negative differences between the BVs of Cr, Pb, Cu, Zn, and Ni and the reference area or the reference soil BVs, slight differences were observed of about <10%.

The HPHP is bounded by the mainstream of the Shaying River and can be divided into two main parts according to soil types, namely the northern (Fluvisols and Cambisols) and southern part (Planosols and Anthrosols). The BVs of soil Hg, As, Cd, Cr, Pb, Cu, Zn, and Ni were 0.066, 4.11, 0.130, 56.72, 20.97, 23.31, 59.21, and 24.03 mg·kg^−1^ in the southern part and 0.061, 7.45, 0.129, 51.92, 18.96, 22.72, 66.96, and 27.16 mg·kg^−1^ in the northern part, respectively.

Besides the BVs of Hg and Cd, there were significant and highly significant differences in the BVs of soil heavy metals between the southern and northern parts of the HPHP. The BVs of Cu in the southern part were significantly higher than those observed in the northern part, while those of As, Zn, and Ni in the northern part were significantly higher than those observed in the southern part. In contrast, the BVs of soil Cr and Pb in the northern part were significantly lower than those revealed in the southern part of the HPHP.

## Figures and Tables

**Table 1:** Descriptive statistics of the soil heavy metal contents after removing outliers (mg·kg^−1^).

HMs	Min	Max	AM	GM	Med	SD	GSD	AMD	CV/%
Hg (*n* = 694)	0.001	0.152	0.070	0.054	0.064	0.041	2.390	0.033	58.57
As (*n* = 848)	1.70	11.25	6.43	5.92	6.67	2.41	1.54	1.96	37.48
Cd (*n* = 682)	0.076	0.183	0.129	0.126	0.129	0.027	1.237	0.020	20.93
Cr (*n* = 729)	35.11	71.65	53.24	52.45	53.13	9.11	1.19	6.64	17.11
Pb (*n* = 778)	14.02	25.54	19.77	19.56	19.67	2.89	1.16	2.32	14.12
Cu (*n* = 613)	14.15	32.77	23.30	22.87	23.05	4.45	1.21	3.26	19.10
Zn (*n* = 550)	40.43	87.87	64.00	62.88	64.04	11.82	1.21	8.74	18.47
Ni (*n* = 753)	16.12	36.36	26.25	25.74	26.08	5.08	1.22	3.85	19.35

**Table 2:** Distribution of soil heavy metal data after removing outliers.

Heavy Metals		Skewness Test			Kurtosis Test			K-S	Q-Q	Distribution
		*S*	*Z_s_*	Distribution	*K*	*Z_k_*	Distribution			
Hg	Natural values	0.311	3.444	skewed	−1.059	5.724	normal	skewed	skewed	skewed
	Log-transformed values	−1.825	19.624	skewed	5.229	28.625	skewed	skewed	skewed	skewed
As	Natural values	0.033	0.393	normal	−1.072	6.381	skewed	skewed	skewed	skewed
	Log-transformed values	−0.644	7.667	skewed	−0.536	3.109	skewed	skewed	skewed	skewed
Cd	Natural values	0.050	0.532	normal	−0.855	4.572	skewed	normal	normal	normal
Cr	Natural values	0.092	1.022	normal	−0.239	1.320	normal	normal	normal	normal
Pb	Natural values	0.074	0.841	normal	−0.964	5.509	skewed	skewed	skewed	skewed
	Log-transformed values	−0.159	1.807	normal	−0.900	5.143	skewed	skewed	skewed	skewed
Cu	Natural values	0.204	2.061	skewed	−0.788	4.000	skewed	skewed	skewed	skewed
	Log-transformed values	−0.140	1.414	normal	−0.765	3.883	skewed	normal	normal	normal
Zn	Natural values	0.030	0.288	normal	0.753	3.620	skewed	normal	normal	normal
Ni	Natural values	0.056	0.628	normal	−0.882	4.955	skewed	normal	normal	normal

**Table 3:** BVs for soil heavy metals and their 95% confidence intervals (mg·kg^−1^).

Heavy Metals	Order Statistics							BVs	95% Confidence Intervals
	5%	10%	25%	50%	75%	90%	95%		
Hg	0.012	0.022	0.035	0.064	0.105	0.131	0.141	0.064 (med)	0.012–0.130
As	2.62	3.06	4.24	6.67	8.32	9.71	10.35	6.67 (med)	2.75–10.59
Cd	0.085	0.093	0.109	0.129	0.149	0.166	0.175	0.129 (AM)	0.075–0.183
Cr	38.29	40.48	46.52	53.13	59.82	66.40	68.24	53.24 (AM)	35.02–71.46
Pb	15.10	16.00	17.44	19.67	22.08	23.81	24.53	19.67 (med)	15.03–24.31
Cu	16.50	17.54	19.81	23.05	26.46	29.61	31.11	22.87 (GM)	15.66–33.39
Zn	43.85	46.51	55.39	64.04	73.00	79.62	83.89	64.00 (AM)	40.36–87.64
Ni	17.80	19.21	22.44	26.08	30.33	33.33	34.90	26.25 (AM)	16.09–36.41

**Table 4:** Comparison of the soil heavy metal BVs between the different regions in China (mg·kg^−1^).

Heavy Metals	This Study	Soils in Henan Province [8]		Fluvo-Aquic Soil in Henan Province [9]		Fluvo-Aquic Soil in China [8]	
		BVs	Degree of Variation (%)	BVs	Degree of Variation (%)	BVs	Degree of Variation (%)
Hg	0.064	0.031	51.56 (Extremely strong)	0.027	57.81 (Extremely strong)	0.032	50.00 (strong)
As	6.67	10.9	−63.43 (Extremely strong)	9.4	−40.93 (strong)	9.3	−39.43 (strong)
Cd	0.129	0.073	43.41 (strong)	0.065	49.61 (strong)	0.085	34.11 (strong)
Cr	53.24	62.5	−17.39 (moderate)	58.9	−10.63 (moderate)	64.8	−20.17 (moderate)
Pb	19.67	19.1	2.90 (weak)	20.1	−2.19 (weak)	20.6	−4.73 (weak)
Cu	22.87	19.2	16.05 (moderate)	−	−	22.9	−3.45 (weak)
Zn	64.00	58.4	8.75 (weak)	−	−	67.8	−5.94 (weak)
Ni	26.25	26.1	0.57 (weak)	25.5	2.86 (weak)	28.1	−7.05 (weak)

**Table 5:** Differences in the heavy metal BVs between the southern and northern parts of the Huang-Huai-Hai Plain of Henan province.

Heavy Metals	Regions	Sample Size After Removing Outliers	Distribution Types	BVs (mg·kg^−1^)	95% Confidence Intervals (mg·kg^−1^)	Statistical Significance
Hg	Southern	261	Skewness	0.066 (Med)	0.003–0.144	Non-significant (*p* = 0.758)
	Northern	426	Skewness	0.061 (Med)	0.003–0.119	
As	Southern	286	Skewness	4.11 (Med)	1.23–6.99	Highly significant (*p* = 0.000)
	Northern	514	Skewness	7.45 (Med)	5.15–9.75	
Cd	Southern	234	Normal	0.130 (AM)	0.092–0.168	Non-significant (*p* = 0.445)
	Northern	427	Normal	0.129 (AM)	0.069–0.189	
Cr	Southern	303	Normal	56.72 (AM)	34.96–78.48	Highly significant (*p* = 0.000)
	Northern	440	Normal	51.92 (AM)	35.22–68.62	
Pb	Southern	298	Log-normal	20.97 (GM)	15.98–27.68	Highly significant (*p* = 0.000)
	Northern	490	Log-normal	18.96 (GM)	14.04–25.60	
Cu	Southern	236	Normal	23.31 (AM)	15.71–30.91	Significant (*p* = 0.038)
	Northern	356	Normal	22.72 (AM)	13.82–31.62	
Zn	Southern	213	Log-normal	59.21 (GM)	41.12–85.26	Highly significant (*p* = 0.000)
	Northern	343	Log-normal	66.69 (GM)	45.68–97.37	
Ni	Southern	296	Normal	24.03 (AM)	13.63–34.07	Highly significant (*p* = 0.000)
	Northern	472	Normal	27.16 (AM)	17.04–37.28	

## Data Availability

Data are contained within the article.

## Supplementary Materials

The following supporting information can be downloaded at: https://www.mdpi.com/article/10.3390/toxics13020093/s1, Figure S1: Soil pH distribution map; Table S1: Setting procedure for automatic digestion of soil samples; Table S2: X2 ICP MS instrument parameters; Table S3: AFS Instrument parameters; Table S4: Analysis results of reference materials (GSS-2); Table S5: The LODs/LOQs of elements.
