# Supplementary material for: Background Values of Soil Heavy Metals in the Huang-Huai-Hai Plain in Henan Province, China
Source: Toxics. 2025 Jan 26;13(2):93. doi: 10.3390/toxics13020093 (PMC11860217; doi:10.3390/toxics13020093)
Supplement: Supplementary file 1 [file toxics-13-00093-s001.zip › toxics-3402988-supplementary.pdf]

Background values of soil heavy metals in the Huang-Huai-Hai Plain in Henan Province, China

Yuling Jiang[1], Jianhua Ma[2,3*], Yuanbo Wang[1], Yahan Yang[1]

[1] *School of Geographic Sciences, Xinyang Normal University, Xinyang 464000, China*

[2] *College of Geography and Environmental Science, Henan University, Kaifeng 475004, China*

[3] *Key Laboratory of Geospatial Technology for the Middle and Lower Yellow River Regions (Henan University), Ministry of Education, Kaifeng 475004, China*

1. Detailed procedure for digestion of soil determination of HMs content

1） Cu, Zn, Cd, Pb, Cr, Ni and other elements in soil samples were pretreated by ST-60 (Beijing Pritac) automatic digestion instrument. Weigh 0.1000g (accurate to 0.1mg) soil sample into the polytetrafluoroethylene digestion tube and moisten it with a small amount of water (including the two blank digestion tubes of the sample). The setting procedure for digestion of soil sample was shown in Table S1.

Table S1 Setting procedure for automatic digestion of soil samples

Serial number	procedure	Add volume (mL)	Heating temperature (℃)	Time (min)	Matters needing attention
1	Add HNO_3	5	/		
2	Oscillation	/		2	
3	Heat		120	60	
4	Cooling			10	
5	Add HF	2.5	/		The soil sample was fully infiltrated
6	Oscillation			2	
7	Heat		140	60	
8	Cooling			10	

9	Add $HCLO_4$	1	/		
10	Oscillation	/	2		
11	Heat	/	160	60	The acid system and soil sample were mixed evenly
12	Heat	/	180	60	
13	Cooling			30	
14	Heat		50	1	Improve the solubility of chromium in digestion solution
15	HNO_3: Water (1:1)	1			
16	Oscillation	/		3	Fully dissolve the filterable residue
17	Constant volume	/			Set volume to 50mL
18	Cap and shake vigorously	/			Full dissolution
19	Standing	/			Settling a small amount of insoluble residue

2）Pretreatment of As and Hg in soil samples were performed by heating in water bath. Weigh 0.5000g sample into a 50 ml colorimetric tube. Add a small amount of water to moisten the sample, followed by 5mL of aqua regia (3:1 mixture of hydrochloric acid and nitric acid). Seal the tube and mix thoroughly. Heat in a water bath at 70°C until it reaches 90°C, then maintain this temperature for 2 hours. Remove and allow to cool.

The sample digestion process utilized super-pure grade reagents (HNO_3, HF, $HCLO_4$, and HCL) obtained from Sinopharm Chemical Reagent Co., Ltd., China. $\rho_{HNO_3} = 1.42g/mL$; $\rho_{HF} = 1.49g/mL$; $\rho_{HCLO_4} = 1.68g/mL$; $\rho_{HCL} = 1.19g/mL$。

2. The operating conditions of ICP-MS and AFS

The operating conditions of ICP-MS are as follows:

Table S2 X2 ICPMS instrument parameters

Parameter	Configuration or parameter	Parameter	Configuration or parameter
Atomizer	Glass concentric atomizer	Standard mode parameter	
Cloud chamber	Conical impact ball fog chamber	Six level bar bias Hex Bias	0
Torch tube	Quartz torch tube	Four pole bias Pole Bias	0
Conical mouth	Xt interface	Focusing lens voltage Focus	12
Injection rate	0.8ml/min	L3	-190
Atomizing gas velocity	0.92ml/min	ICP power	1400W
Auxiliary gas flow rate	0.7ml/min	Cooling gas	13L/min

The operating conditions of AFS are as follows:

Table S3 Instrument parameters

Parameter	As	Hg
Negative high voltage/V	280	280
Lamp current/mA	40	20
Atomizer height/mm	8	8
Carrier gas flow rate /(mL/min)	400	400
Shielding gas flow rate /(mL/min)	900	900
Counting method	Peak area	Peak area
Delay time/s	2	2
Integration time/s	10	10

3. Analysis results of reference materials

The external standard method served as the primary analytical approach in this experiment, and 1000 μg/mL external standard solution Rh was provided by China

Nonferrous Metals and Electronic Materials Analysis and Test Center. The standard soil sample (GSS-2) was purchased from the Center of National Standard Reference Material of China. The addition concentrations of the soil reference material and the recoveries of different elements were shown in the Table 4.

Table S4 Analysis results of reference materials (GSS-2)

Element	Reference material	Certified concentration (μg/g)	Measured value	Plus standard value (μg/g)	Standard recovery rate /%
As	GBW07402(GSS-2)	13.7±1.2	13.5	10.0	105.5
Cd		0.071±0.014	0.065	0.2	92
Cr		47±4	45.4	20	91.3
Cu		16.3±0.9	16.5	20	108.9
Hg		0.015±0.003	0.016	0.30	99.1
Ni		19.4±1.3	19.1	20	96.6
Pb		20±3	21	20	106.6
Zn		42±3	40	20	85.8

4. The LODs/LOQs of elements

LODs and LOQs are estimated from concentrations corresponding to 3 and 10 times the standard deviation (σ) of the blank concentration of the element measured by this method. In this study, a set of repeated measurements were carried out on the corresponding blank digestion solution, and the determined concentration value of each element (mg/L) was converted into the corresponding content value (mg/kg), and the results were shown in Table 5.

Table S5 The LODs/LOQs of elements

Heavy metals	Limit of detection (mg/kg)	Limit of quantification(mg/kg)
Cd	0.0003	0.0009
Pb	0.004	0.015
As	0.001	0.003
Cr	0.004	0.014

Cu	0.004	0.015
Zn	0.07	0.25
Ni	0.004	0.015
Hg	0.002	0.007

5. Determination of soil pH

Weigh 10.0g of air-dried soil through a 2-mm sieve into a 50mL beaker, add 25mL deionized water, stir with a glass rod for 1 min to make the soil particles fully dispersed, and place for 30 min for determination. Pour the soil superliquant into a 20ml small beaker, insert the electrode into the liquid to be tested, gently shake the beaker to remove the water film on the electrode and promote its rapid balance, let it stand for a moment, press the reading switch, and record the pH when the reading is stable (pH change within 5s is less than 0.02). Release the reading switch to take out the electrode, wash with water, and use the filter paper strip to absorb the water, then the second sample can be measured.

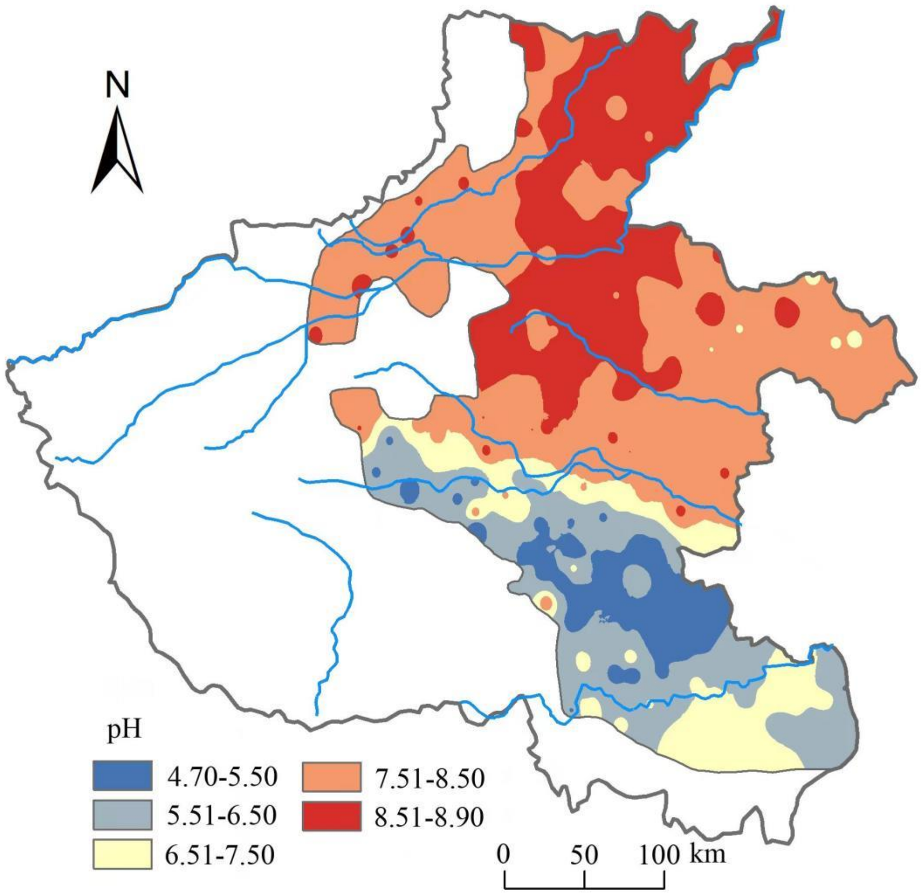

Figure.S1 Soil pH distribution map
